# 2316. SARS-CoV-2 N-antibody Seropositivity in Healthcare Personnel without a Known History of COVID-19

**DOI:** 10.1093/ofid/ofad500.1938

**Published:** 2023-11-27

**Authors:** Sajal K Tiwary, Carrie O’Neal, Kate Peacock, Rachel Bosserman, Candice Cass, Mostafa Amor, Karl Hock, Meghan Wallace, David Macdonald, Olivia G Arter, Kelly Alvarado, Lucy Vogt, Henry B Stewart, Daniel Park, Claire Dalton, Carey-Ann Burnham, Christopher Farnsworth, Jennie H Kwon

**Affiliations:** Washington University in St. Louis, St. Louis, Missouri; Washington University in St. Louis, St. Louis, Missouri; Washington University in St. Louis, St. Louis, Missouri; Washington University School of Medicine, St. Louis, Missouri; Washington University, St. Louis, Missouri; Washington University in St. Louis, St. Louis, Missouri; Washington University School of Medicine, St. Louis, Missouri; Washington university School of Medicine, Hillsboro, Missouri; Washington University in St. Louis, St. Louis, Missouri; Washington University in St. Louis, St. Louis, Missouri; Washington University in St. Louis, St. Louis, Missouri; Washington University, St. Louis, Missouri; Washington University School of Medicine in St. Louis, Indianapolis, Indiana; Washington University School of Medicine, St. Louis, Missouri; Washington University in St. Louis, St. Louis, Missouri; Pattern Bioscience, Saint Louis, MO; Washington University in St. Louis, St. Louis, Missouri; Washington University - School of Medicine, St. Louis, MO

## Abstract

**Background:**

Healthcare personnel (HCP) are at risk for SARS-CoV-2 exposure in both the healthcare and community setting, and the rate of unconfirmed infection in HCP is unknown. Our objective was to measure the rate of SARS-CoV-2 seropositivity in HCP with no history of COVID-19, but who work with COVID-19 patients and/or specimens.

**Methods:**

This was a prospective cohort study of HCP at a tertiary care academic medical center in St. Louis, MO. Asymptomatic HCP who work with COVID-19 patients and/or specimens and no history of known COVID-19 were enrolled between September 22, 2020 to October 10, 2021. Follow-up visits were conducted between December 1, 2020 to March 3, 2022, at a 1-6 month interval from enrollment. At enrollment, participants completed a survey with questions about demographics, employment, and pre-existing medical conditions. At both visits, a blood sample was obtained for SARS-CoV-2 anti-N testing and participants provided information about SARS-CoV-2 exposures, social distancing behaviors, travel and social event history, and symptoms of COVID-19. Odds ratios (OR) with 95% confidence intervals (CI) were calculated to examine associations between N-antibody test results and HCP characteristics.

**Results:**

A total of 727 participants completed an enrollment visit, with 559 also completing a follow-up visit. The median age of HCP was 35 [IQR 30, 47]. The cohort was 71% female and 82% white. At enrollment, 3.7% of HCP had a reactive SARS-CoV-2 anti-N test. HCP who were seropositive at enrollment were more likely to regularly work with COVID-19 patients (OR 2.09; 95% CI, 0.94 - 4.77) and to have had a household exposure to COVID-19 in the past 30 days (OR 7.92, 95% CI, 2.44 - 25.73). At follow-up, 10.2% of HCP were seropositive. Seropositivity at follow-up was associated with exposure to a household member with COVID-19 (OR 9.50 [5.02, 17.96]) as well as other community exposures to COVID-19 (OR 2.90 [1.31, 6.44]).

**Conclusion:**

A small percentage of HCP with no history of known COVID-19 were seropositive for SARS-CoV-2 N-antibody, with this rate increasing later in the pandemic. Exposures to individuals with COVID-19 in and out of the workplace remains a strong risk for seropositivity in HCP. Thus, protection against COVID-19 both in and outside of the healthcare setting remains necessary.

**Disclosures:**

**Carey-Ann Burnham, PhD**, Pattern Bioscience: Stocks/Bonds **Christopher Farnsworth, PhD**, Abbott Laboratories: Grant/Research Support|Abbott Laboratories: Honoraria|Biomerieux: Grant/Research Support|Cepeheid: Grant/Research Support|Roche Diagnostics: Grant/Research Support|Siemens Healthineers: Grant/Research Support|Werfen: Advisor/Consultant

